# Analysis of the physiological load on lumbar vertebrae in patients with osteoporosis: a finite-element study

**DOI:** 10.1038/s41598-022-15241-3

**Published:** 2022-06-29

**Authors:** Sungwook Kang, Chan-Hee Park, Hyunwoo Jung, Subum Lee, Yu-Sun Min, Chul-Hyun Kim, Mingoo Cho, Gu-Hee Jung, Dong-Hee Kim, Kyoung-Tae Kim, Jong-Moon Hwang

**Affiliations:** 1 Precision Mechanical Process and Control R&D Group, Korea Institute of Industrial Technology, Jinju, 52845 Korea; 2 Department of Rehabilitation Medicine, Kyungpook National University Hospital, Daegu, 41944 Korea; 3 Department of Neurosurgery, Korea University Anam Hospital, Seoul, 02841 Korea; 4 Department of Rehabilitation Medicine, Kyungpook National University Chilgok Hospital, Daegu, 41404 Korea; 5 Department of Rehabilitation Medicine, School of Medicine, Kyungpook National University, Daegu, 41944 Korea; 6 Department of Neurosurgery, School of Medicine, Kyungpook National University, Daegu, 41944 Korea; 7 Department of Orthopaedic Surgery, Gyeongsang National University, College of Medicine and Gyeongsang National University Changwon Hospital, Changwon, 51472 Korea; 8 Department of Orthopaedic Surgery, Institute of Health Science, Research Institute of Clinical Medicine, Gyeongsang National University School of Medicine and Hospital, Jinju, 52727 Korea

**Keywords:** Biological techniques, Biophysics, Computational biology and bioinformatics, Neurology

## Abstract

This study aims to investigate the difference in physiological loading on the spine in three different motions (flexion–extension, lateral bending, and axial rotation) between osteoporotic and normal spines, using finite element modelling. A three-dimensional finite element (FE) model centered on the lumbar spine was constructed. We applied two different material properties of osteoporotic and normal spines. For the FE analysis, three loading conditions (flexion–extension, lateral bending, and axial rotation) were applied. The von Mises stress was higher on the nucleus pulposus at all vertebral levels in all movements, in the osteoporosis group than in the normal group. On the annulus fibrosus, the von Mises stress increased at the level of L3–L4, L4–L5, and L5–S in the flexion–extension group and at L4–L5 and L5–S levels in the lateral bending group. The values of two motions, flexion–extension and lateral bending, increased in the L4 and L5 cortical bones. In axial rotation, the von Mises stress increased at the level of L5 of cortical bone. Additionally, the von Mises stress increased in the lower endplate of L5–S and L4–L5 in all movements, especially lateral bending. Even in the group with no increase, there was a part that received increased von Mises stress locally for each element in the three-dimensional reconstructed view of the pressure distribution in color. The von Mises stress on the lumbar region in the three loading conditions, was greater in most components of osteoporotic vertebrae than in normal vertebrae and the value was highest in the nucleus pulposus. Considering the increase in the measured von Mises stress and the local increase in the pressure distribution, we believe that these results can contribute to explaining discogenic pain and degeneration.

## Introduction

Osteoporosis is a worldwide health problem of considerable magnitude. It is the most common cause of fractures and is estimated to affect 1.5 million individuals each year^1^. Moreover, the risk of fractures due to trauma increases in patients with preexisting osteoporosis^2^. Osteoporotic fractures are classified as vertebral or non-vertebral and occur most commonly in the hip, wrist, and humerus^3^. In Europe, according to the European Vertebral Osteoporosis Study (EVOS), the prevalence of osteoporosis is 12.2% among males and 12% in those aged between 50 and 79 years^4^. The annual incidence is 1%, 2%, and 3% in women aged 65, 75, and 85 years, respectively. In males over 50 years of age, the prevalence ranges between 5.7 and 6.8/1,000 person/years, which is equivalent to approximately half of that seen in women^5^. Osteoporosis and poor bone health, similar to other chronic diseases, are increasingly becoming a burden on society.

Vertebral compression fractures are the most common type of osteoporotic fracture^6^. These may cause low back pain and limitation of daily life owing to pain, which can become chronic^7^. Management of chronic pain in patients with osteoporosis involves a combination of pharmacological and nonpharmacological therapies and exercises to improve axial stability^8^. However, since it is difficult to measure the difference in pressure within spinal structures, experimental studies on the difference in pressure distribution between normal and osteoporotic vertebrae have rarely been conducted.

We believe that the physical properties of the osteoporotic spine differ from those of the normal spine. As a result, the vertebral load is expected to be higher in the osteoporotic spine. This study aims to investigate the difference in physiological load on the spine with three different motions between osteoporotic and normal spines, using finite element modelling. To our knowledge, this is the first study evaluating this research hypothesis.

## Methods

A three-dimensional (3D) finite element (FE) analysis was carried out to investigate the effects of various load modes (flexion–extension, lateral bending, and axial rotation) on the lumbar spine and disc, in normal and osteoporotic patients. The distribution of the calculated von Mises stress was observed by applying loads to a 3D finite element model, including the lumbar vertebrae and disc. This work was supported by the Biomedical Research Institute grant, Kyungpook National University Hospital (2021).

### Development of the FE model

**Figure 1:**
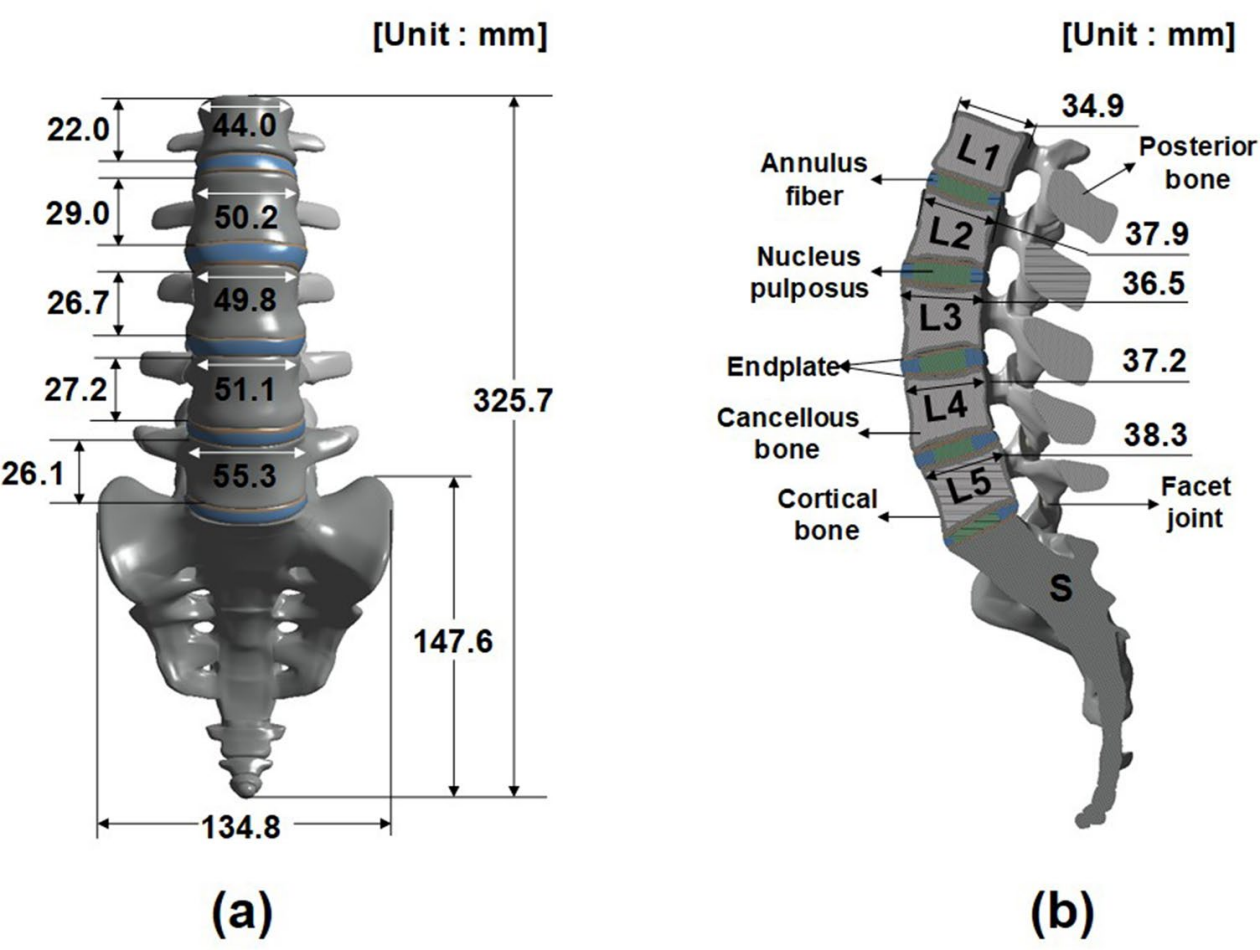
Dimension of finite element model (**a**) Front view, (**b**) Section view.

A 3D FE model centered on the lumbar spine was constructed^9–11^. In this study, a finite element model was created for men in their mid-30 s with a height of 175 cm. Digital data of the human body were collected from the KISTI (Korea Institute of Science and Technology Information) and used by agreement. There was no need for IRB approval. The KISTI provided the Korean human information (such as CT, MR, serially sectioned image, segmented image and 3D image) since 2000 and many kinds of Korean human data were prepared and serviced to the users in domestic and abroad. The specific dimensions of the model are presented in Fig. 1. Although there is a size difference in the male and female models, it is considered that the tendency to interpret the results will be similar because the components are the same. Compared with the 75 lumbar dimensions measured by Wolf et al., 67 data are within the experimental data range. The remaining 8 data are within the maximum error range of 2.1 mm or less. This means that the dimensions of the finite element model presented in this study are appropriate^12^. It comprised the sacrum, L1 to L5 lumbar vertebrae (including the cortical and cancellous bones and posterior element), intervertebral discs (including nucleus pulposus and annulus fibrosus), endplates, and facet joints, also as shown in Fig. 1. The posterior elements consisted of the pedicles, lamina, facets (articular process), and transverse and spinous processes. The end plate was a bilayer of cartilage and bone that separated the intervertebral discs from the adjacent vertebrae. The 3D FE model was completed by converting the surface model into a solid model using CT data. Model conversion was performed using the 3D computer-aided design (CAD) softwares, CATIA (Dassault Systèmes, Vélizy-Villacoublay, France) and ANSYS SpaceClaim, (SpaceClaim Corporation, Concord, MA, USA).

### Mesh and material properties for the FE model

The mesh for the FE analysis was created using the Static Structural module of the ANSYS Workbench. The element size was determined through a sensitivity analysis of the mesh. As the element type, a second-order tetrahedron (10 nodes) was used. When the human body moves out of the elastic range, bones fracture or a disc problem occurs. In this case, the plastic properties are required for the analysis. However, in this study, the material properties of the vertebral body in the elastic range are required because the stress received by the vertebral body is analyzed after applying the value of the load that can occur when a person generally moves. Therefore, for FE analysis, material properties such as the elastic modulus and Poisson’s ratio of the components, are required^13^. These material properties differ in normal and osteoporotic patients; patients with osteoporosis have lower bone density than normal individuals. In the case of elastic modulus of components related to bone is smaller in osteoporotic patients than in normal individuals. In contrast, the modulus of elasticity of intervertebral discs is relatively larger in osteoporotic patients than in normal individuals. Information on the mesh size and material properties for the FE model is summarized in Table 1^14–16^. One standard analysis case was set to determine the initial mesh size. Normal material properties and von-Mises stresses in L4–L5 Nucleus pulposus during flexion–extension loading were compared according to mesh size. First, the mesh size of the entire model was set from 1 to 5 mm at intervals of 1 mm, and then von-Mises stress was calculated for the above reference analysis case. As a result of the analysis, the stress difference between 1 and 2 mm was 1.55%, so 2 mm was set as the standard mesh size. Based on this, while increasing the mesh size of each part in Table 1 at intervals of 1 mm and comparing the stress with the standard mesh size (2 mm), the mesh size that falls within 2% was finally set as shown in Table 1 (1.78% error compared to the standard mesh size).

**Table 1:** Information of mesh and material properties for the finite element model.

Item	Element size (mm)	Number of nodes	Number of elements	Elastic modulus E (MPa)		Poisson's ratio V		Reference
				Normal	Osteoporosis	Normal	Osteoporosis	
Cortical bone	3	69,667	37,138	12,000	8040	0.3	0.3	^15,16^
Cancellous bone	5	71,381	47,169	200	34	0.25	0.25	^15,16^
Posterior bone	4	37,963	20,990	3,500	2345	0.25	0.25	^15,16^
Endplate	2	30,138	13,503	1,000	670	0.3	0.3	^14,15^
Nucleus pulposus	3	346,396	239,611	1	9	0.49	0.4	^14,15^
Annulus fiber	3	358,484	239,295	4.2	5	0.45	0.45	^14,15^
Facet joint	2	1842	474	11	11	0.4	0.4	^16^

*MPa* megapascal.

### Loading and boundary conditions

**Figure 2:**
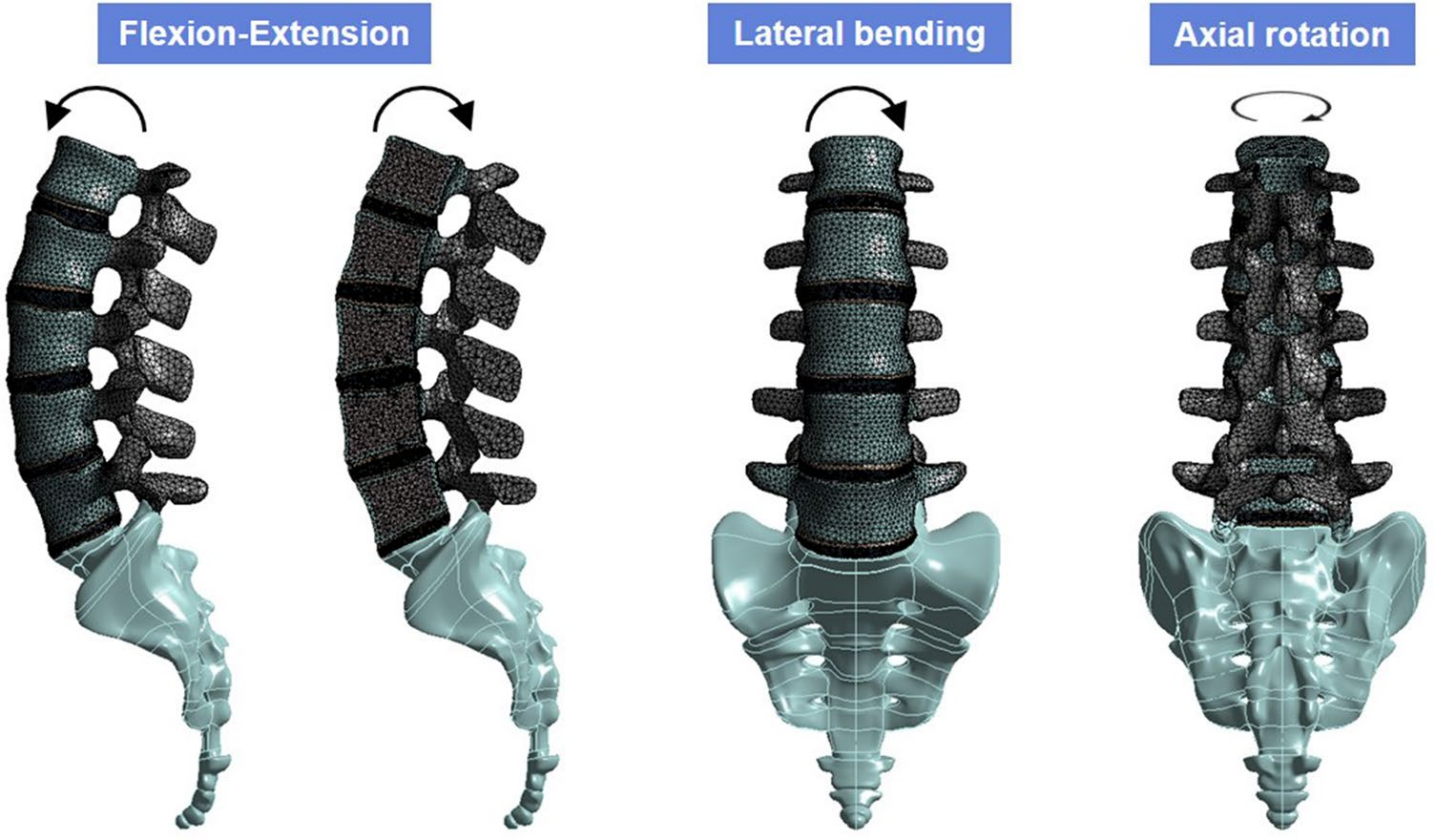
Three loading conditions (flexion–extension, lateral bending, and axial rotation).

For the FE analysis, three loading conditions (flexion–extension, lateral bending, and axial rotation) were applied, as shown in Fig. 2. A moment of 400 N m was applied to the human body structure. The total degrees of freedom of the sacrum were fixed, and bonding contact conditions were applied to each component of the human body so that they did not separate from each other when a load was applied. Referring to the existing research results, the contact condition of each component is assumed to be a bonding contact. Therefore, it is considered to be very difficult to subdivide the contact conditions of each component in practice^17–19^.

## Results

### von Mises stress in the spine FE model

**Figure 3:**
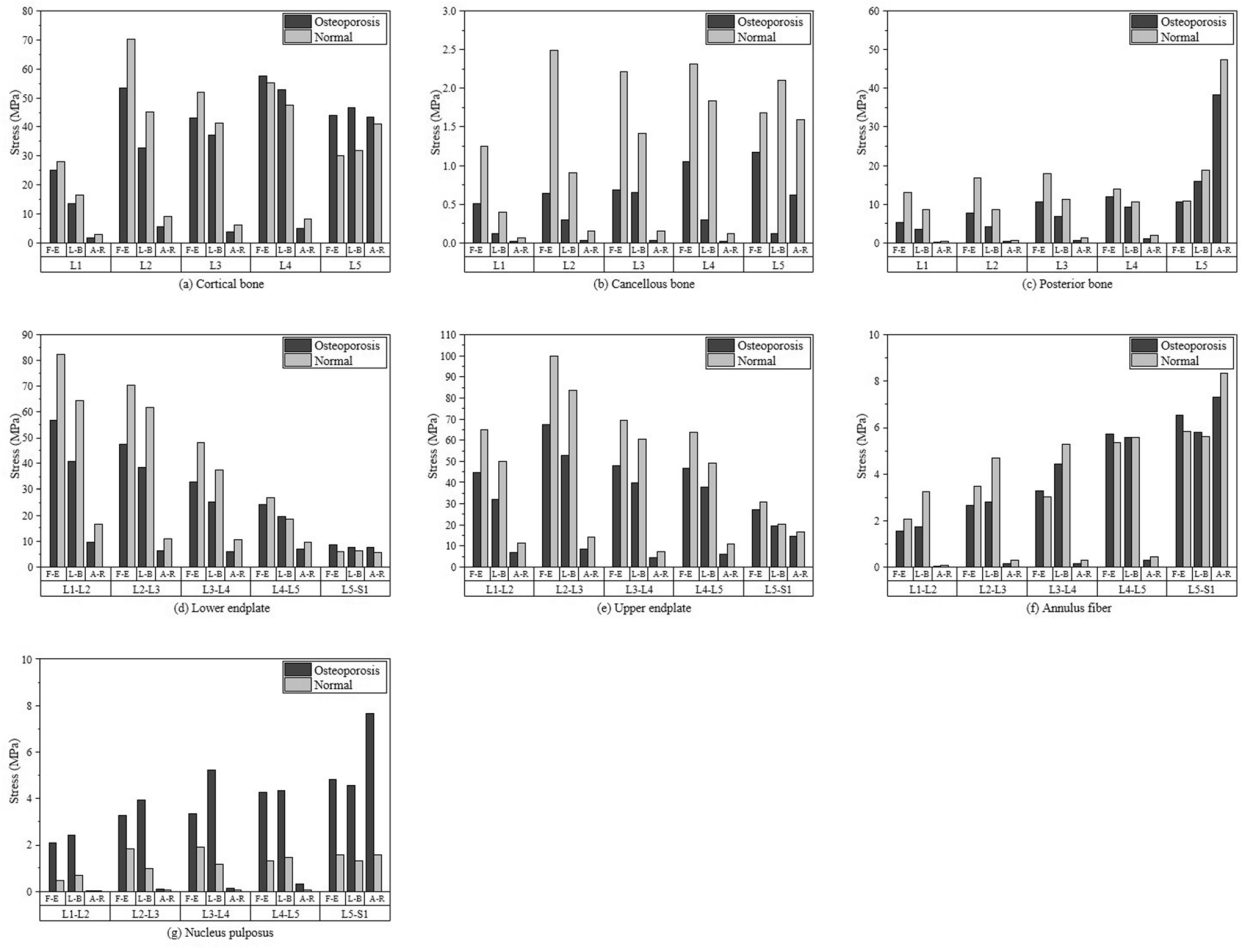
von-Mises stress results for three loading modes which were flexion–extension (F–E), lateral bending (L-B) and axial rotation (A-R).

The von Mises stress was compared with the average of each component volume. Tables 2, 3, 4 and Fig. 3 show the von Mises stress results for each loading mode.

**Table 2:** von-Mises stress results for Flexion–Extension loading mode.

Component	Lumbar level	Osteoporosis (A) (MPa)	Normal (B) (MPa)	Loading ratio (A − B)/B × 100 (%)
Cortical bone	L1	25.126	28.122	− 10.65
	L2	53.361	70.227	− 24.02
	L3	43.054	52.126	− 17.40
	L4	57.693	55.173	4.57*
	L5	43.967	30.076	46.19*
Cancellous bone	L1	0.51006	1.2454	− 59.04
	L2	0.64338	2.4965	− 74.23
	L3	0.68258	2.2162	− 69.20
	L4	1.0499	2.3183	− 54.71
	L5	1.1709	1.6815	− 30.37
Posterior bone	L1	5.3673	13.053	− 58.88
	L2	7.6343	16.807	− 54.58
	L3	10.621	17.928	− 40.76
	L4	11.871	13.915	− 14.69
	L5	10.534	10.699	− 1.54
Lower endplate	L1–L2	56.732	82.265	− 31.04
	L2–L3	47.543	70.456	− 32.52
	L3–L4	33.017	48.089	− 31.34
	L4–L5	24.117	26.97	− 10.58
	L5–S	8.424	5.8443	44.14*
Upper endplate	L1–L2	44.762	64.95	− 31.08
	L2–L3	67.358	100.08	− 32.70
	L3–L4	47.912	69.273	− 30.84
	L4–L5	46.778	63.812	− 26.69
	L5–S	26.971	30.933	− 12.81
Annulus fiber	L1–L2	1.5629	2.0654	− 24.33
	L2–L3	2.6575	3.4548	− 23.08
	L3–L4	3.2907	3.0178	9.04*
	L4–L5	5.7342	5.3602	6.98*
	L5–S	6.5444	5.818	12.49*
Nucleus pulposus	L1–L2	2.1013	0.46321	353.64*
	L2–L3	3.2781	1.8385	78.3*
	L3–L4	3.3553	1.8947	77.09*
	L4–L5	4.2502	1.3132	223.65*
	L5–S	4.8262	1.5877	203.97*

*MPa* megapascal.

*Percentage of increased load in osteoporosis than normal.

**Table 3:** von-Mises stress results for Lateral bending loading mode.

Component	Lumbar level	Osteoporosis (A) (MPa)	Normal (B) (MPa)	Loading ratio (A − B)/B × 100 (%)
Cortical bone	L1	13.497	16.516	− 18.28
	L2	32.68	45.26	− 27.79
	L3	37.049	41.368	− 10.44
	L4	52.804	47.511	11.14*
	L5	46.602	31.986	45.69*
Cancellous bone	L1	0.12077	0.39291	− 69.26
	L2	0.29689	0.90154	− 67.07
	L3	0.6554	1.4151	− 53.69
	L4	0.29689	1.8355	− 83.83
	L5	0.12077	2.1061	− 94.27
Posterior bone	L1	3.418	8.6089	− 60.30
	L2	4.2622	8.6113	− 50.50
	L3	6.8846	11.261	− 38.86
	L4	9.1897	10.523	− 12.67
	L5	15.874	18.713	− 15.17
Lower endplate	L1–L2	40.946	64.309	− 36.33
	L2–L3	38.612	61.885	− 37.61
	L3–L4	25.25	37.471	− 32.61
	L4–L5	19.487	18.555	5.02*
	L5–S	7.6198	6.0725	25.48*
Upper endplate	L1–L2	32.16	49.854	− 35.49
	L2–L3	52.787	83.705	− 36.94
	L3–L4	39.689	60.355	− 34.24
	L4–L5	37.619	49.207	− 23.55
	L5–S	19.313	20.299	− 4.86
Annulus fiber	L1–L2	1.7252	3.2563	− 47.02
	L2–L3	2.8048	4.6763	− 40.02
	L3–L4	4.4374	5.293	− 16.16
	L4–L5	5.5855	5.5839	0.03*
	L5–S	5.7893	5.6254	2.91*
Nucleus pulposus	L1–L2	2.4159	0.69101	249.62*
	L2–L3	3.9118	0.9813	298.63*
	L3–L4	5.2183	1.1694	346.24*
	L4–L5	4.3405	1.4543	198.46*
	L5–S	4.5578	1.3158	246.39*

*MPa* megapascal.

*Percentage of increased load in osteoporosis than normal.

**Table 4:** von-Mises stress results for Axial rotation loading mode.

Component	Lumbar level	Osteoporosis (A) (MPa)	Normal (B) (MPa)	Loading ratio (A − B)/B × 100 (%)
Cortical bone	L1	1.7847	2.9347	− 39.19
	L2	5.559	9.1105	− 38.98
	L3	3.6444	6.1652	− 40.89
	L4	4.8083	8.3163	− 42.18
	L5	43.408	41.068	5.7*
Cancellous bone	L1	0.0151	0.0633	− 76.15
	L2	0.0306	0.14743	− 79.24
	L3	0.0313	0.15748	− 80.12
	L4	0.0235	0.11987	− 80.40
	L5	0.61867	1.598	− 61.28
Posterior bone	L1	0.11104	0.28003	− 60.35
	L2	0.31113	0.59648	− 47.84
	L3	0.64636	1.3479	− 52.05
	L4	0.94167	1.9366	− 51.38
	L5	38.26	47.385	− 19.26
Lower endplate	L1–L2	9.6145	16.477	− 41.65
	L2–L3	6.1934	10.881	− 43.08
	L3–L4	5.9779	10.619	− 43.71
	L4–L5	7.0101	9.6956	− 27.70
	L5–S	7.5692	5.3671	41.03*
Upper endplate	L1–L2	6.7984	11.463	− 40.69
	L2–L3	8.3617	14.076	− 40.60
	L3–L4	4.2539	7.4382	− 42.81
	L4–L5	6.0596	10.761	− 43.69
	L5–S	14.466	16.651	− 13.12
Annulus fiber	L1–L2	0.0386	0.0688	− 43.90
	L2–L3	0.12801	0.26926	− 52.46
	L3–L4	0.14739	0.2947	− 49.99
	L4–L5	0.26848	0.43696	− 38.56
	L5–S	7.301	8.3341	− 12.40
Nucleus pulposus	L1–L2	0.0303	0.0152	99.34*
	L2–L3	0.10799	0.055	96.35*
	L3–L4	0.12821	0.058	121.05*
	L4–L5	0.30552	0.0769	297.3*
	L5–S	7.6572	1.587	382.5*

*MPa* megapascal.

*Percentage of increased load in osteoporosis than normal.

We compared the von Mises stress on each component at each level of the lumbar spine between the osteoporosis and normal groups. The ratio of the von Mises stress difference, expressed as a percentage, was calculated using the formula (A − B)/B × 100 (%), where A and B represent the osteoporotic and normal groups, respectively. A positive value denotes that the osteoporosis group takes more von Mises stress than the normal group.

Comparing the osteoporosis and normal groups in the flexion–extension action group, the values of L4 and L5 cortical bones as per the above formula were 4.57% and 46.19%, respectively, indicating that the von Mises stress increased by this value in osteoporosis. The cancellous and posterior bones and the upper endplate, did not differ between the two groups. In the lower endplate of L5–S, the von Mises stress increased by 44.14% in osteoporosis. In addition, L3–L4, L4–L5, and L5–S annulus fibers in the osteoporosis group had von Mises stresses higher by 9.04%, 6.98%, and 12.49%, respectively. At all levels from L1–L2 to L5–S, the von Mises stresses on the nucleus pulposus increased by 353.64%, 78.3%, 77.09%, 223.65%, and 203.97%, respectively.

In the lateral bending motion group, the von Mises stress in the osteoporosis group increased to 11.14% and 45.69% in the cortical bone of L4 and L5, respectively, and to 5.02% and 25.48% in the lower endplate of the L4–L5 and L5–S, respectively. In addition, the von Mises stresses on L4–L5 and L5–S annulus fibers increased slightly by 0.03% and 2.91%, respectively, and across all levels, the von Mises stress on the nucleus pulposus increased by 249.62%, 298.63%, 346.24%, 198.46%, and 246.39%, in the osteoporosis group.

Finally, in the axial rotation group, the von Mises stress on the cortical bone of L5 in the osteoporosis group increased by 5.7% and that on the lower endplate of L5-S increased by 41.03%. As in the other motions, the von Mises stress in the nucleus pulposus at all lumbar levels, increased by 99.34%, 96.35%, 121.05%, 297.3%, and 382.5%, respectively. There were no differences between the two groups with respect to the annulus fibrosus, including the cancellous and posterior bones and the upper endplate.

All three motions, flexion–extension, lateral bending and axial rotation, had the largest absolute values in the nucleus pulposus at all lumbar spine levels, suggesting that it bore a large von Mises stress in osteoporosis. In particular, the values were large in the flexion–extension and lateral bending motions and relatively small during axial rotation.

**Figure 4:**
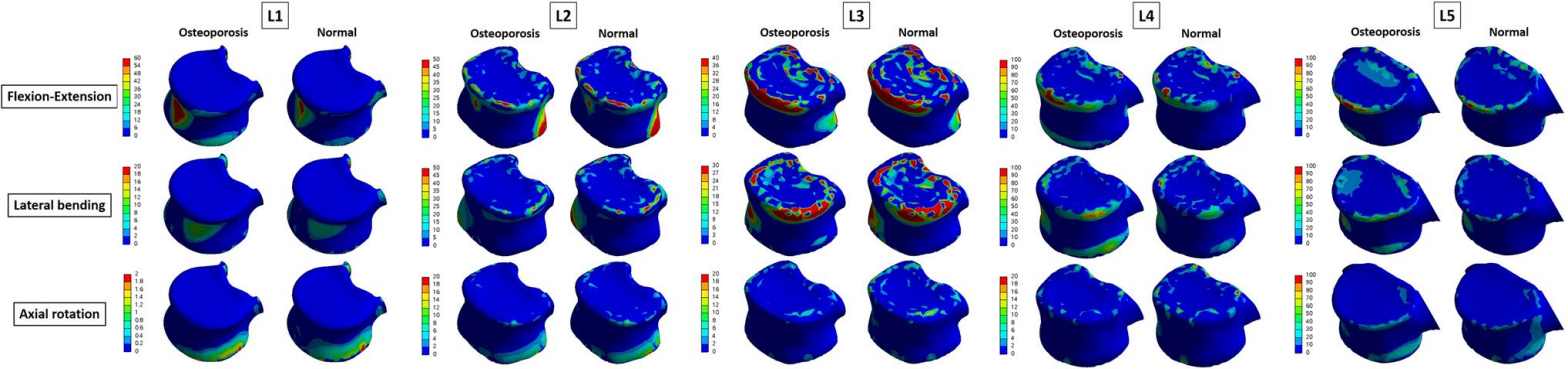
Von-Mises Stress Results at cortical bone in three different motions (Unit: MPa).

**Figure 5:**
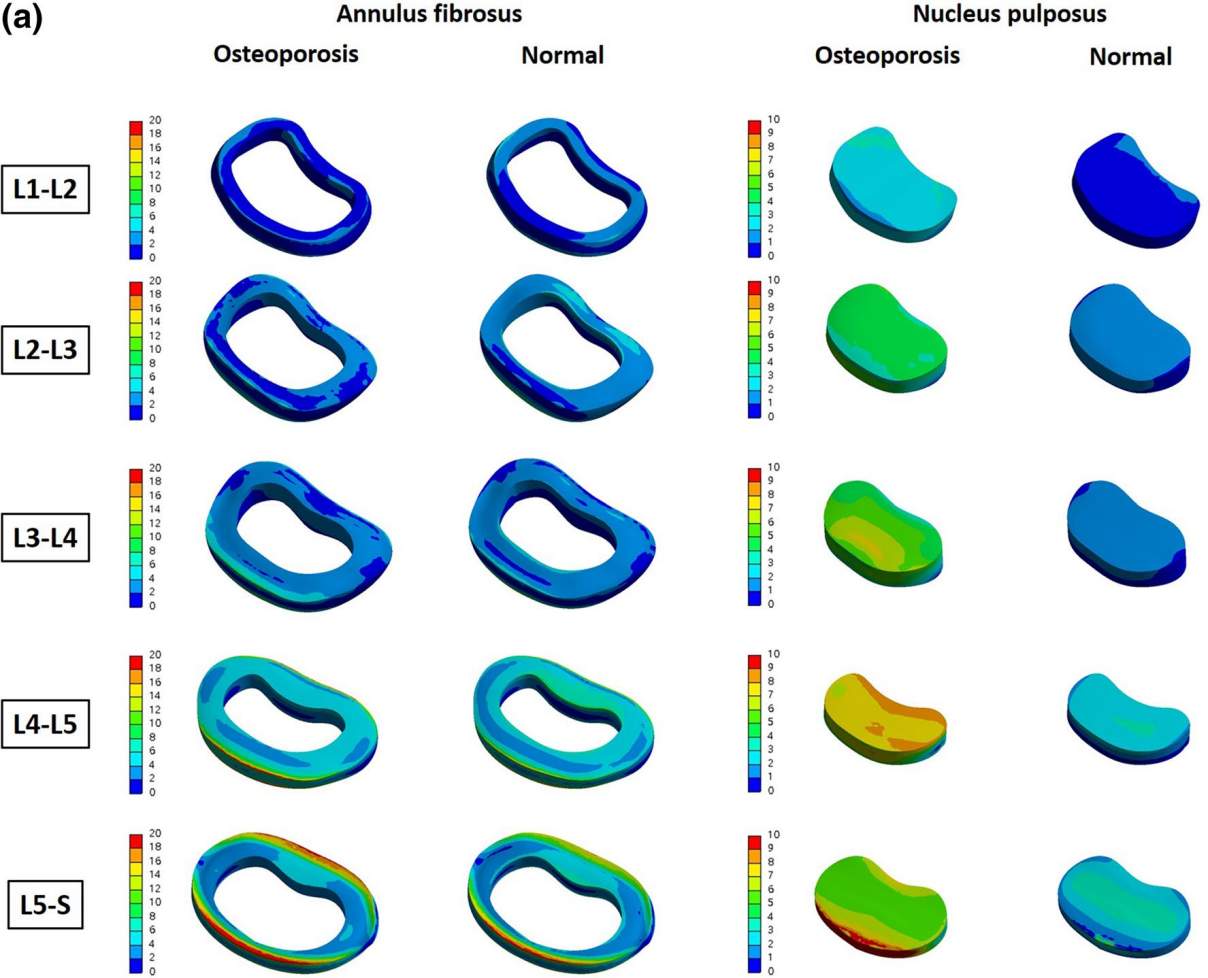

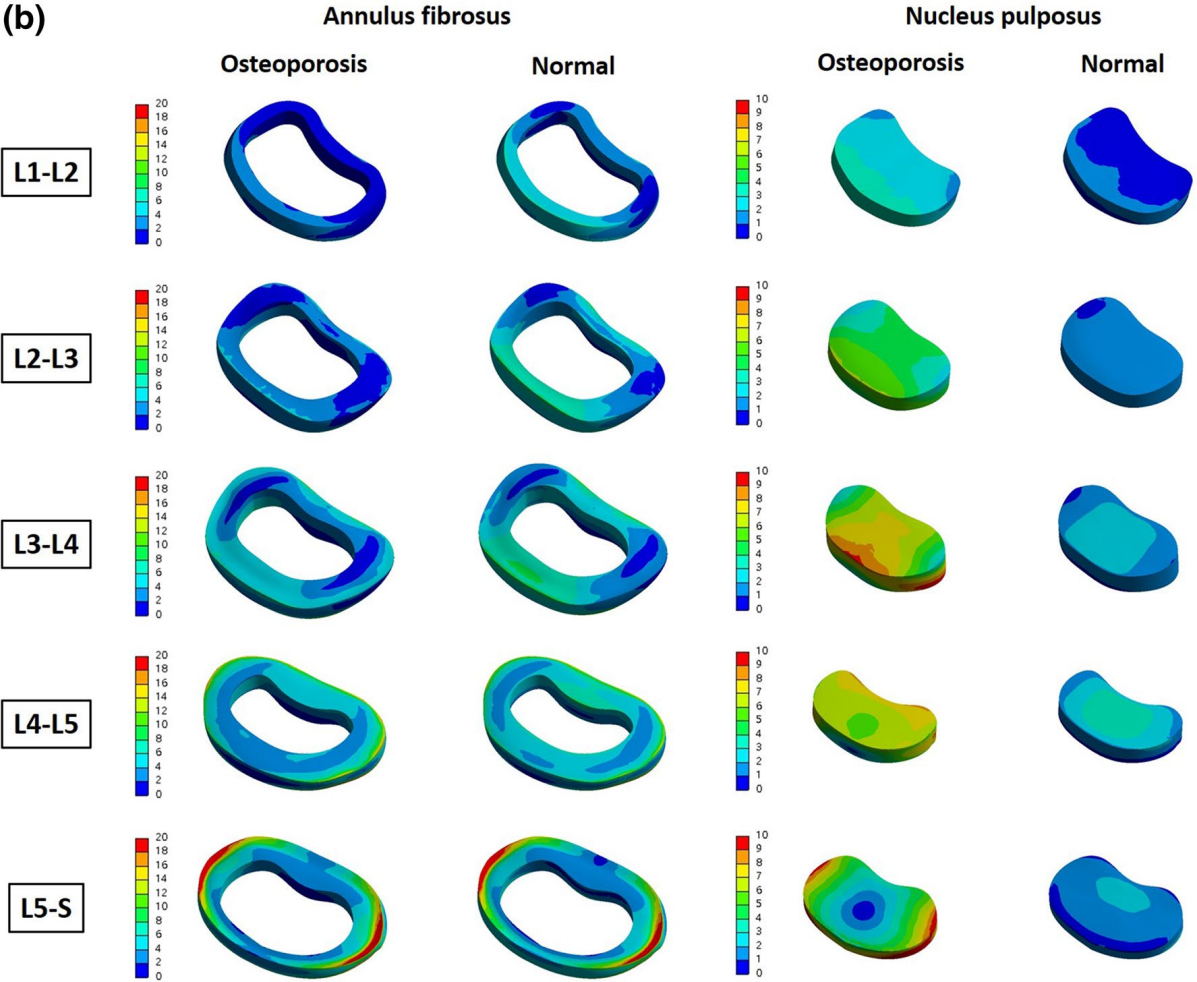

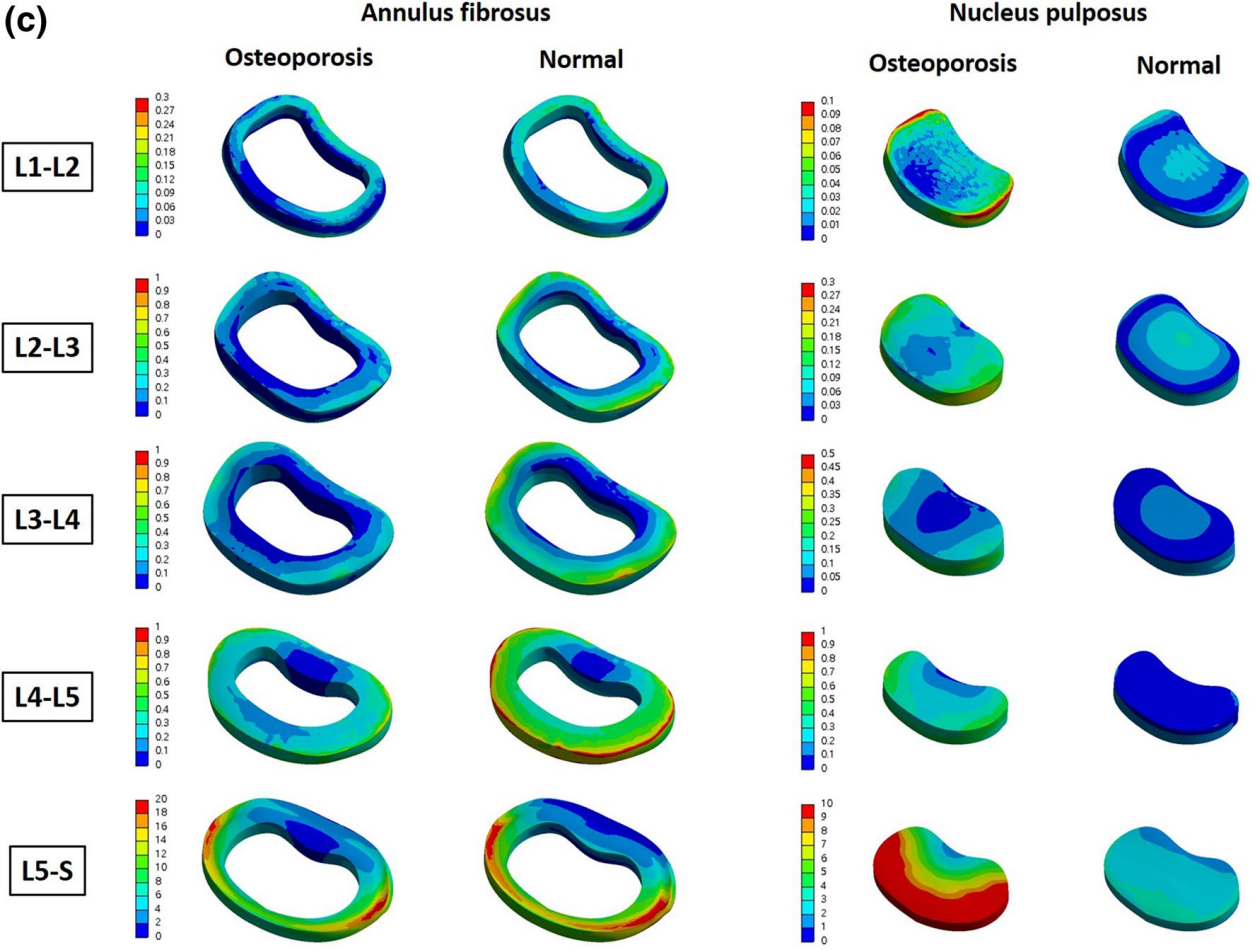
(**a**) von Mises stress distribution on the intervertebral disc in flexion–extension loading mode. As a result of analysis in flexion–extension motion, the stress was concentrated in the annulus fibrosus of L3–L4, L4–L5 and L5–S, and in the nucleus of whole level of lumbar (Unit: MPa). (**b**) von Mises stress distribution on the intervertebral disc in lateral bending loading mode. As a result of analysis in lateral bending motion, the stress was concentrated in the annulus fibrosus of L4–L5 and L5–S, and in the nucleus of whole level of lumbar (Unit: MPa). (**c**) von Mises stress distribution on the intervertebral disc in axial rotation loading mode. As a result of analysis in axial motion, the stress was mainly concentrated in the nucleus of whole level of lumbar (Unit: MPa).

We also reconstructed the above results in 3D and expressed the difference in von Mises stress distribution of the entire part including the surface, in color. Figure 4 shows an analysis of the cortical bone. Looking at L4 and L5 in the above results, which show that more von Mises stress is received in osteoporosis, it was confirmed that the value of the von Mises stress corresponding to red at a specific part of the interface, was high. In the results of the table above (even from L1 to L3, which had a positive value), there was a specific part in which the von Mises stress corresponding to red was increased. Figure 5 shows the von Mises stress distribution for the intervertebral discs, including the annulus fibrosus and nucleus pulposus. Regarding the cortical bone, it can be seen from the image that there is a partially increased part even in the group where the calculated von Mises stress value does not appear to have increased.

Additionally, equivalent strain values were analyzed as shown in Tables 5, 6 and 7. In the material properties of Table 1, the elastic modulus of bone for osteoporotic patients is small compared to normal people, and the disc is large. If the modulus of elasticity is small, the resistance to external force is relatively weak and the strain is large. From Tables 5, 6 and 7, it can be seen that the bone equivalent strain of osteoporotic patients is greater than that of normal people. In particular, it can be seen that the equivalent strain difference occurs a lot in the cancellous bone where the elastic modulus difference is large. On the other hand, in the case of discs (especially Nucleus pulposus), the elastic modulus of osteoporotic patients is greater than that of normal individuals, so it can be seen that the equivalent strain in the disc is small in osteoporotic patients.

**Table 5:** Equivalent strain results for Flexion–Extension loading mode.

Component	Lumbar level	Osteoporosis (A)	Normal (B)	Loading ratio (A − B)/B × 100 (%)
Cortical bone	L1	0.0053	0.0043	24.44
	L2	0.0099	0.0089	10.95
	L3	0.0077	0.0065	18.05
	L4	0.0097	0.0068	43.20
	L5	0.0071	0.0035	100.67
Cancellous bone	L1	0.0155	0.0068	129.54
	L2	0.0198	0.0133	48.58
	L3	0.0217	0.0127	71.13
	L4	0.0316	0.0122	158.30
	L5	0.0350	0.0086	308.98
Posterior bone	L1	0.0025	0.0040	− 38.43
	L2	0.0035	0.0052	− 31.83
	L3	0.0049	0.0056	− 11.42
	L4	0.0056	0.0044	28.09
	L5	0.0049	0.0034	47.22
Lower endplate	L1–L2	0.1331	0.1300	2.34
	L2–L3	0.1072	0.1059	1.23
	L3–L4	0.0781	0.0763	2.42
	L4–L5	0.0499	0.0409	22.15
	L5–S	0.0129	0.0060	114.06
Upper endplate	L1–L2	0.1379	0.1344	2.64
	L2–L3	0.1482	0.1467	1.05
	L3–L4	0.1061	0.1025	3.56
	L4–L5	0.1039	0.0964	7.80
	L5–S	0.0578	0.0490	17.94
Annulus fiber	L1–L2	0.3173	0.4977	− 36.25
	L2–L3	0.5382	0.8297	− 35.13
	L3–L4	0.6680	0.7270	− 8.12
	L4–L5	1.1710	1.3000	− 9.92
	L5–S	1.3482	1.4257	− 5.44
Nucleus pulposus	L1–L2	0.2337	0.4645	− 49.69
	L2–L3	0.3645	0.7404	− 50.77
	L3–L4	0.3734	0.6757	− 44.73
	L4–L5	0.4753	1.3243	− 64.11
	L5–S	0.5430	1.6178	− 66.44

**Table 6:** Equivalent Strain results for Lateral bending loading mode.

Component	Lumbar level	Osteoporosis (A)	Normal (B)	Loading ratio (A − B)/B × 100 (%)
Cortical bone	L1	0.0029	0.0027	9.11
	L2	0.0059	0.0057	3.65
	L3	0.0063	0.0051	22.93
	L4	0.0084	0.0054	55.26
	L5	0.0072	0.0033	116.54
Cancellous bone	L1	0.0039	0.0025	59.12
	L2	0.0092	0.0051	80.78
	L3	0.0202	0.0083	145.28
	L4	0.0310	0.0095	228.30
	L5	0.0413	0.0107	287.24
Posterior bone	L1	0.0016	0.0026	− 41.06
	L2	0.0020	0.0027	− 25.93
	L3	0.0032	0.0035	− 8.90
	L4	0.0043	0.0033	30.49
	L5	0.0074	0.0058	26.57
Lower endplate	L1–L2	0.0965	0.1026	− 5.92
	L2–L3	0.0854	0.0918	− 6.97
	L3–L4	0.0570	0.0569	0.22
	L4–L5	0.0369	0.0266	38.36
	L5–S	0.0116	0.0062	86.46
Upper endplate	L1–L2	0.1037	0.1086	− 4.47
	L2–L3	0.1112	0.1186	− 6.25
	L3–L4	0.0841	0.0855	− 1.69
	L4–L5	0.0826	0.0731	13.00
	L5–S	0.0377	0.0297	26.80
Annulus fiber	L1–L2	0.3474	0.7796	− 55.43
	L2–L3	0.5666	1.1207	− 49.44
	L3–L4	0.9000	1.2738	− 29.35
	L4–L5	1.1386	1.3531	− 15.85
	L5–S	1.1829	1.3688	− 13.58
Nucleus pulposus	L1–L2	0.2686	0.6916	− 61.17
	L2–L3	0.4348	0.9825	− 55.74
	L3–L4	0.5803	1.1735	− 50.55
	L4–L5	0.4841	1.4656	− 66.97
	L5–S	0.5084	1.3319	− 61.83

**Table 7:** Equivalent Strain results for Axial rotation loading mode.

Component	Lumbar level	Osteoporosis (A)	Normal (B)	Loading ratio (A − B)/B × 100 (%)
Cortical bone	L1	0.0005	0.0006	− 11.51
	L2	0.0012	0.0013	− 10.18
	L3	0.0008	0.0009	− 14.39
	L4	0.0010	0.0012	− 15.28
	L5	0.0061	0.0041	50.26
Cancellous bone	L1	0.0006	0.0005	23.37
	L2	0.0011	0.0009	15.18
	L3	0.0013	0.0012	6.95
	L4	0.0009	0.0009	7.80
	L5	0.0184	0.0081	127.73
Posterior bone	L1	0.0001	0.0001	− 36.32
	L2	0.0001	0.0002	− 21.99
	L3	0.0003	0.0004	− 28.69
	L4	0.0004	0.0006	− 27.48
	L5	0.0176	0.0146	20.33
Lower endplate	L1–L2	0.0228	0.0262	− 12.92
	L2–L3	0.0145	0.0171	− 14.98
	L3–L4	0.0139	0.0165	− 15.98
	L4–L5	0.0158	0.0165	− 3.92
	L5–S	0.0115	0.0056	107.30
Upper endplate	L1–L2	0.0208	0.0235	− 11.42
	L2–L3	0.0192	0.0218	− 11.82
	L3–L4	0.0099	0.0116	− 14.78
	L4–L5	0.0157	0.0187	− 16.10
	L5–S	0.0296	0.0273	8.27
Annulus fiber	L1–L2	0.0081	0.0168	− 51.71
	L2–L3	0.0262	0.0651	− 59.81
	L3–L4	0.0300	0.0710	− 57.81
	L4–L5	0.0541	0.1048	− 48.34
	L5–S	1.4723	2.0025	− 26.48
Nucleus pulposus	L1–L2	0.0035	0.0152	− 77.01
	L2–L3	0.0120	0.0553	− 78.23
	L3–L4	0.0143	0.0582	− 75.50
	L4–L5	0.0340	0.0771	− 55.94
	L5–S	0.8523	1.5932	− 46.50

## Discussion

In this study, FE analysis was used to compare the von Mises stresses on the lumbar spine and intervertebral discs when various load-modes were applied to normal and osteoporotic spines. The cortical bone had the greatest von Mises stress, that is, the von Mises stress was found to be concentrated on the cortical bone that supports the body structure. In particular, the largest von Mises stress occurs at L4 and L5, and it can be seen that in patients with osteoporosis, the stress is greater than that in normal persons at the corresponding positions (L4 and L5). In the nucleus pulposus, it was confirmed that patients with osteoporosis had 77.09–382.50% greater von Mises stress than those without. However, because the results shown in Tables 2, 3 and 4 are based on the total value for each part, there is a limitation in that the von Mises stress increase in specific parts cannot be reflected, as shown in Figs. 4 and 5. In other words, even if it has a positive value, it can be compensated for by a sufficiently negative value, meaning that more von Mises stress is applied if a specific part is coordinated. Therefore, the table value and 3D von Mises stress distribution in color should be simultaneously compared, and the fact that a positive value does not indicate less von Mises stress should not be overlooked. As shown in Figs. 4 and 5, the fact that the osteoporosis group had a higher von Mises stress on the surface bordering other adjacent sites may help explain the mechanism by which the following phenomena occur. First, it will be helpful to explain the mechanisms by which (a) osteophyte develops in the spine secondary to degeneration, and (b) intervertebral disc degeneration results in discogenic pain^20–22^. In other words, in osteoporotic patients, the von Mises stress on the nucleus pulposus is more concentrated than that in normal individuals, which can accelerate disc degeneration^23,24^.

Furthermore, several studies have shown that chronic back pain is caused by spinal deformity or kyphosis in patients with osteoporosis; furthermore, chronic low back pain tends to improve with pharmacological treatment of osteoporosis. According to our experimental model, this result can be explained by the difference in stress applied to each part in osteoporotic patients. These results further suggest a potential reduction in the incidence of vertebral fractures after proper treatment for osteoporosis in such patients. According to previous studies, pharmacological treatment is effective in preventing osteoporotic fractures^25^. Based on our research, we believe that the pharmacological treatment of osteoporosis will help prevent fractures by affecting the physical properties of each element of the spine analyzed above, leading to a reduction in the von Mises stress on each element.

This study has several limitations. First, the incidence of osteoporotic vertebral fractures is highest in the lower thoracic (T11 and T12) and upper lumbar (L1) vertebrae^7^. However, this study was conducted only on the lumbar spine, which, while advantageous for controlling variables, excludes the thoracic spine. Second, our FE model did not include tendons, nerves, ligaments, and muscles, and did not reflect the variability of these structures between the two groups and for each motion. However, the effect of the structures was minimized through comparative analysis, with the results obtained by including the structures above^26^. In other words, the muscles and other tissues associated with the vertebral column differed from individual to individual rather than from osteoporosis. It was considered that even osteoporosis patients could not conclude that they lack muscle. And the difference in the physical properties of bones and discs between normal people and osteoporosis patients has already been reported by other existing studies. However, in the case of muscles and ligaments, no data on material properties have been reported for normal people and osteoporotic patients. Therefore, in this study, except for muscles and ligaments with uncertain material properties, the study was conducted only considering the effects (differences) on the bones and discs of normal people and osteoporosis patients. If differences in muscle or ligament properties between normal people and osteoporosis patients are reported in the future, research can be conducted taking this into account. Currently, it is limited to obtain the ligament properties of normal people and osteoporosis patients due to the limitations of the experiment. For this reason, in this study, the muscles and other tissues were not set as variables, and they were not included in the finite element model. Therefore, only the difference in the physical properties of bones and discs between normal people and osteoporosis patients was used as a variable and the analysis was performed. Therefore, further analysis is needed to study the influence of muscles and other tissues in osteoporosis patients and normal people, and it is considered that studies that implement and simulate a finite element model are needed in the future. In addition, the differences in intervertebral disc height or physical properties were not reflected in the performance of each motion. Third, because this study is a model-based analysis, it is limited in that it does not reflect the actual clinical characteristics and risk factors of osteoporosis. Although it was based on the physical properties of each component in osteoporosis, as presented in Table 1, it is difficult to accurately represent the condition of the individual spine in clinical practice. As mentioned above, structural differences such as bony spur or disc degeneration, are often accelerated when osteoporosis is already present, but it is difficult to fully reflect these factors in a single model. In addition, it does not reflect the risk factors related to osteoporosis, such as age, low body weight, glucocorticoid therapy, current cigarette smoking, excessive alcohol consumption, previous fracture, and secondary osteoporosis^27,28^.

## Conclusion

In conclusion, the von Mises stress on the lumbar region in the three motions-flexion–extension, lateral bending, and axial rotation-was greater in most components of osteoporotic vertebrae, and the von Mises stress was highest in the nucleus pulposus. According to the three-dimensionally reconstructed pressure distribution diagram, even in areas where the von Mises stress did not increase, these factors may help explain the discogenic pain or degeneration occurring in osteoporosis, via a local increase in pressure in some regions.

## Data Availability

The datasets analyzed during the current study are available from the corresponding author on reasonable request.

## References

[1] Office of the Surgeon General (2004). Bone Health and Osteoporosis: A Report of the Surgeon General.

[2] Mackey DC (2007). High-trauma fractures and low bone mineral density in older women and men. JAMA.

[3] Stone KL (2003). BMD at multiple sites and risk of fracture of multiple types: Long-term results from the Study of Osteoporotic Fractures. J. Bone Miner. Res..

[4] O'Neill T (1994). Reproducibility of a questionnaire on risk factors for osteoporosis in a multicentre prevalence survey: The European Vertebral Osteoporosis Study. Int. J. Epidemiol..

[5] Felsenberg D (2002). Incidence of vertebral fracture in Europe: Results from the European Prospective Osteoporosis Study (EPOS). J. Bone Mineral Res..

[6] Genant HK (1999). Interim report and recommendations of the World Health Organization task-force for osteoporosis. Osteoporos. Int..

[7] Rosen, H. N. & Walega, D. R. Osteoporotic thoracolumbar vertebral compression fractures: Clinical manifestations and treatment. https://www.uptodate.com/contents/osteoporotic-thoracolumbar-vertebralcompression-fractures-clinical-manifestations-and-treatment. (2017).

[8] Paolucci T, Saraceni VM, Piccinini G (2016). Management of chronic pain in osteoporosis: Challenges and solutions. J. Pain Res..

[9] Jain P, Khan MR (2022). Comparison of novel stabilisation device with various stabilisation approaches: A finite element based biomechanical analysis. Int. J. Artif. Organs.

[10] Jain P, Rana M, Biswas JK, Khan MR (2020). Biomechanics of spinal implants: A review. Biomed. Phys. Eng. Express.

[11] Jain P, Khan MR (2021). Selection of suitable pedicle screw for degenerated cortical and cancellous bone of human lumbar spine: A finite element study. Int. J. Artif. Organs.

[12] Wolf A, Shoham M, Michael S, Moshe R (2001). Morphometric study of the human lumbar spine for operation–workspace specifications. Spine.

[13] Kang S (2021). The effects of paraspinal muscle volume on physiological load on the lumbar vertebral column: A finite-element study. Spine.

[14] Byun D-H, Shin DA, Kim J-M, Kim S-H, Kim H-I (2012). Finite element analysis of the biomechanical effect of coflex™ on the lumbar spine. Korean J. Spine.

[15] Cho A-R, Cho S-B, Lee J-H, Kim K-H (2015). Effect of augmentation material stiffness on adjacent vertebrae after osteoporotic vertebroplasty using finite element analysis with different loading methods. Pain Physician.

[16] Park WM, Kim K, Kim YH (2013). Effects of degenerated intervertebral discs on intersegmental rotations, intradiscal pressures, and facet joint forces of the whole lumbar spine. Comput. Biol. Med..

[17] Deoghare AB, Kashyap S, Padole PM (2013). Investigation of biomechanical behavior of lumbar vertebral segments with dynamic stabilization device using finite element approach. 3D Res..

[18] Guo H (2021). A finite element study on the treatment of thoracolumbar fracture with a new spinal fixation system. BioMed. Res. Int..

[19] Schmidt H, Midderhoff S, Adkins K, Wilke H-J (2009). The effect of different design concepts in lumbar total disc arthroplasty on the range of motion, facet joint forces and instantaneous center of rotation of a L4–5 segment. Eur. Spine J..

[20] Fujii K (2019). Discogenic back pain: Literature review of definition, diagnosis, and treatment. JBMR Plus.

[21] Peng B (2005). The pathogenesis of discogenic low back pain. J. Bone Joint Surg..

[22] Pye SR (2007). Lumbar disc degeneration: Association between osteophytes, end-plate sclerosis and disc space narrowing. Ann. Rheum. Dis..

[23] Harada A, Okuizumi H, Miyagi N, Genda E (1998). Correlation between bone mineral density and intervertebral disc degeneration. Spine.

[24] Tsouknidas A, Sarigiannidis SO, Anagnostidis K, Michailidis N, Ahuja S (2015). Assessment of stress patterns on a spinal motion segment in healthy versus osteoporotic bony models with or without disc degeneration: A finite element analysis. Spine J..

[25] Jin Y-Z, Lee JH, Xu B, Cho M (2019). Effect of medications on prevention of secondary osteoporotic vertebral compression fracture, non-vertebral fracture, and discontinuation due to adverse events: A meta-analysis of randomized controlled trials. BMC Musculoskelet. Disord..

[26] Mimura M (1994). Disc degeneration affects the multidirectional flexibility of the lumbar spine. Spine.

[27] Lewiecki, E. M. & Schmader, K. E. Osteoporotic fracture risk assessment. https://www.uptodate.com/contents/osteoporotic-fracture-riskassessment. (2013).

[28] Pisani P (2016). Major osteoporotic fragility fractures: Risk factor updates and societal impact. World J. Orthop..

